# Assessing post-cue exposure craving and its association with amount wagered in an optional betting task

**DOI:** 10.1556/JBA.2.2013.011

**Published:** 2013-06-14

**Authors:** Lisham Ashrafioun, John Kostek, Erin Ziegelmeyer

**Affiliations:** Department of Psychology, Bowling Green State University, Bowling Green, OH, USA

**Keywords:** craving, gambling, university students, blackjack

## Abstract

*Background and aims:* The current study was designed to assess the impact of wins and losses in simulated blackjack on craving to gamble and to assess the extent to which this craving was associated with actual wagering in an optional gambling task. *Methods:* Participants were undergraduates attending a large Midwestern university in the United States. They completed the Gambling Urge Scale (GUS) and then were randomized to either a condition in which they would win 15 hands of blackjack (Win condition; *n* = 41) or lose 15 hands (Lose condition; *n* = 37) out of a total of 20 hands. After playing blackjack and completing several additional questionnaires, participants had the chance to wager their $5 compensation for the opportunity to win $50. *Results:* GUS scores increased significantly following blackjack, regardless of condition. We also found that post-blackjack craving was significantly associated with the amount participants wagered in the optional betting task, such that greater craving was associated with higher amount wagered. *Conclusions:* These findings provide further support for the construct validity of the GUS, provide novel findings regarding the effects of wins and losses when gambling, and provide evidence of an association between craving and a behavioral betting task.

## Introduction

The prevalence and problems associated with gambling are a concern in many university settings. For example, Barnes, Welte, Hoffman and Tidwell (2010) found that nearly 75% of a sample of over 1000 university students had gambled in the past year and 6% reported having experienced at least two problems associated with gambling. These prevalence rates are important given the various social, economic and educational consequences of such gambling (e.g., loss of money intended for academic-related activities, missing class, damaging social networks; Barnes et al., 2010; Shaffer, Hall & Vander Bilt, 1999; Stinchfield, Hanson & Olson, 2006; Welte, Barnes, Tidwell & Hoffman, 2008).

Theoretical and empirical evidence suggests that craving is an important factor in the elicitation and maintenance of problem gambling (Blaszczynski & Nower, 2002; Hodgins & el-Guebaly, 2004; Ladouceur, Sylvain & Gosselin, 2007; Moran, 1970; Oei & Gordon, 2008; Shaffer, 1999; Sharpe, 2002). For example, Blaszczynski and Nower (2002) posited that intermittent wins and relief from depression, anxiety and or boredom contribute to the development of habitual patterns of gambling. As cognitive biases related to the probability of winning and habitual patterns of gambling become established, efforts to restrain gambling may cause craving to gamble. In addition, internal cues (e.g., negative affect, boredom) and external cues (e.g., sight of playing cards, sounds of a slot machine) that are repeatedly paired with gambling can themselves elicit urges to gamble (Sharpe, 2002).

Several researchers have examined the effect of exposure to gambling-related cues on craving (Ashrafioun, McCarthy & Rosenberg, 2012; Kushner et al., 2007, 2008; Sodano & Wulfert, 2010; Wulfert, Maxson & Jardin, 2009; Young & Wohl, 2009). For example, Ashrafioun et al. (2012) found that university student gamblers reported an increase in craving to gamble following exposure to both gambling-related photographs and a gambling-related imagery script. Furthermore, Wulfert et al. (2009) found that a community sample of pathological gamblers reported stronger urges when presented with video clips representing their preferred mode of gambling compared to their non-preferred mode.

Several studies have assessed whether craving to gamble changed following manipulations of wins and losses. For example, Kushner et al. (2008) found that frequent gamblers reported an increase in craving after observing and playing blackjack and that craving was higher following wins compared to losses. In addition, Sodano and Wulfert (2010) showed that active pathological gamblers had increased heart rates and reported greater craving after viewing a brief video depicting winning money compared to losing money.

These studies suggest that gambling-related cue exposure and simulated gambling can influence the extent to which one experiences craving. However, these studies have assessed craving using single-item rating scales. Although single-item measures have the advantage of being administered quickly, they do not assess the variety of experiences that could be indicative of craving, allow for the calculation of reliability statistics, and respondents may have difficulty indicating their craving with a single numeral (Ashrafioun & Rosenberg, 2012; Rosenberg, 2009).

Researchers have developed multi-item measures to assess craving to gamble that have advantages of being relatively short and having strong psychometric support (see Ashrafioun & Rosenberg, 2012 for a review of measures to assess craving to gamble). For example, Raylu and Oei (2004) designed the Gambling Urge Scale (GUS) to assess current craving to gamble. To develop the GUS, Raylu and Oei rephrased items from the Alcohol Urge Questionnaire (Bohn, Krahn & Staehler, 1995) to ask respondents to rate the degree to which they agreed with statements describing the quality or intensity of current urges to gamble. Because the GUS comprises only six items, it is especially suitable for use as a screening instrument and repeated measurement in both assessment and therapy sessions. Researchers have also found support for elements of its construct, convergent, criterion, and discriminant validities in university, community, and clinical samples of gamblers (Ashrafioun et al., 2012; Raylu & Oei, 2004; Smith, Pols, Battersby & Harvey, 2013).

Despite the psychometric support for the GUS, there have not been any studies that have assessed the effects of manipulating wins and losses in a simulated gambling task on GUS craving. In addition, it is unclear the extent to which GUS craving is associated with short-term *future* gambling behaviors. In light of the prevalence of gambling among university students, the impact of wins and losses on craving, and limitations of previous research on the validity of the GUS, we designed the present study to accomplish several goals. Firstly, to test the construct validity of the GUS, we wanted to assess the effect of cue exposure on craving. We also manipulated wins and losses for the cue exposure to evaluate the impact of blackjack outcomes on craving. We expected that those randomized to a condition in which participants were programmed to win the majority of their hands would report a greater increase in craving compared to a condition in which participants were programmed to lose the majority of their hands.

Secondly, to assess the extent to which GUS craving is associated with short-term future gambling behaviors, we evaluated the association between post-cue exposure GUS scores and the amount wagered on an optional betting task administered at the end of study participation. We hypothesized that greater craving would be positively associated with the amount wagered on the optional betting task.

## Methods

### Participants and procedures

Following approval from our Institutional Review Board, we recruited undergraduate psychology students from a large Midwestern university using a research session management system website, which included a brief description of the study, eligibility criteria (i.e., understand the basic rules of blackjack and be a current gambler), and compensation opportunities (i.e., research participation credit, $5.00, and two chances to win $20.00). Of the 116 undergraduates who participated in the study, 37 were excluded from further analysis for reporting that they do not currently gamble, which was discovered subsequently. This exclusion left 79 participants for analysis: 42% were female, 47% were freshman, and 73% identified as Caucasian, and 44% identified scratch/lottery as the most preferred mode of gambling. The typical week frequency of gambling was relatively low (*M* = 0.3, *SD* = 0.6, range 0 to 3) and did not differ by preferred mode of gambling, *F*(2,75) = 1.02, *p* < .05. Their ages ranged from 18 to 29 with a mean of 19 years (*SD* = 1.6). Please see Table 1 for additional information regarding the sample’s demographic and gambling history characteristics.

All eligible participants provided verbal informed consent, were seated at a computer in a quiet laboratory room, and completed the pre-cue exposure GUS. Participants were then randomly assigned to the Win condition (*n* = 41), in which they were programmed to win 15 out of 20 hands, or the Lose condition (*n* = 38), in which they were programmed to lose 15 out of 20 hands. After playing blackjack, we re-administered the GUS in addition to a questionnaire that assessed demographics and gambling history characteristics. After participants completed the questionnaires, they were compensated with $5.00 and asked if they would like to wager their compensation for an opportunity to win $50. Participants were then debriefed and thanked for their participation. This study was conducted as part of a larger study that also examined risky decision making (Kostek & Ashrafioun, 2013).

### Measures

*Gambling Urge Scale (GUS).* To assess current craving to gamble, respondents were asked to rate their level of agreement using a seven-point scale (“Completely disagree” to “Completely agree”). Examples of items include “All I want to do now is to gamble”, “It would be difficult to turn down a gamble this minute”, and “I want to gamble so bad that I can almost feel it”. Internal consistency reliabilities in our sample were .93 for pre-cue exposure GUS and .96 for post-cue exposure GUS.

*Demographics and gambling history questionnaire.* We developed this self-report questionnaire for this study to assess demographic information (e.g., age, gender, ethnicity) and gambling history. Gambling history questions included: “Type in how old you were (in years) when you gambled for the first time”, “Type in the amount of money you spend on gambling in a typical week”, “How many days do you gamble during a typical week?” and “What is your choice type of gambling?”

### Blackjack manipulation

All participants played twenty hands of blackjack on the computer starting with a total of 500 points and could wager between five and 25 points per hand. The starting point and betting amounts were selected to ensure participants could not lose all of their points before the final hand. To encourage participants to do well, participants were told that if they scored in the top 50% of participants, they would be entered into a drawing to win $20.

Using E-Prime v.1.2 (Psychology Software Tools, Inc.), we programmed the twenty hands of blackjack such that those in the Win condition won 15 of the 20 hands and those in the Lose condition lost 15 of the 20 hands. Participants in the Win condition finished with an average of 649 points (*SD* = 58) and participants in the Lose condition finished with an average of 371 points (*SD* = 46). Because the winning condition automatically gained more points, the $20 prize was awarded randomly to one of the participants, regardless of condition.

### Final bet

Participants were given five, one-dollar bills as compensation at the end of the study. Prior to leaving the study site, they were informed that they could wager some or all of the compensation for the chance to win $50. They were shown a small clear bag filled with 199 blue and green beads and then shown a single orange bead. They were instructed that if they drew the orange bead out of the bag, they would win the $50. The orange bead was placed in the clear bag in front of the participant and then the clear bag was placed into a black cloth bag so that the beads were no longer visible. Each draw cost participants $1 and participants had the option to draw up to five beads, but they had to pay for and select only one bead at a time with replacement. Therefore, participants could complete their participation with all five of their dollars, none of the five dollars, or an amount in between. No participants won the $50.

## Results

**Figure 1.**
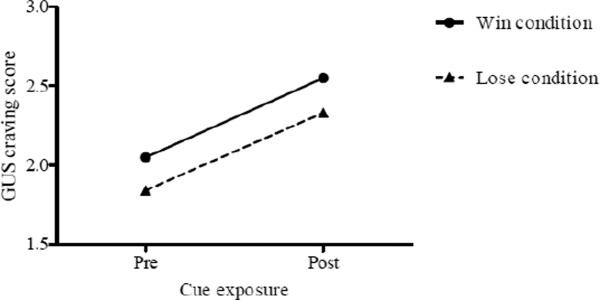
Mean scores on the Gambling Urge Scale pre- and post-cue exposure by blackjack outcome condition

We conducted a two-way mixed model analysis of variance (ANOVA) with time (pre-cue exposure; post-cue exposure) as the within-subjects factor, blackjack outcome condition (win; loss) as the between-subjects factor, and mean post-cue exposure GUS scores as the dependent variable. The ANOVA revealed a significant main effect of time, *F*(1, 77) = 23.65, *p* < .001, partial h^2^ = .24. Specifically, there was a significant increase in craving to gamble from pre-cue exposure (*M* = 1.9, *SD* = 1.1) to post-cue exposure (*M* = 2.4, *SD* = 1.5). Neither the two-way interaction, nor the main effect of condition was significant (see Figure 1). This pattern of findings provides support for the construct validity of the GUS by showing that exposure to simulated blackjack hands elicited craving and that this increase occurred regardless of the outcome of the 20 hands.

**Table 1.** Mean *(SD)* or % of demographics, gambling characteristics, and outcome variables

Characteristics	Mean *(SD)* or % of Win condition (*n* = 41)	Mean *(SD)* or % of Lose condition (*n* = 38)	Mean *(SD)* or % of Total (*n* = 79)
Age (years)	19.1 (1.0)	19.3(1.8)	19.2(1.6)
Gender			
Male	56%	59%	58
Female	44	41	42
Ethnicity			
White/European American	71	76	73
Black/African American	22	16	19
Other	7	8	8
Year in college			
First year	41	53	47
Second year	37	24	30
Third year	12	16	14
Fourth year +	10	8	9
Employment status			
Unemployed	59	53	57
Employed	41	47	44
(part- or full-time)			
Preferred gambling mode			
Lottery/scratch	46	41	44
Cards	37	30	33
Other (e.g., sports, casino games)	17	29	23
Age of 1st gamble	14.8 (3.6)	14.6 (2.8)	15.1 (3.3)
Days gambled per week	0.3 (0.7)	0.5 (0.7)	0.3 (0.6)
Gambling Urge Scale score			
Pre-cue exposure	2.0(1.2)	1.8(1.0)	1.7(1.0)
Post-cue exposure	2.6 (1.5)	2.3 (1.5)	2.2(1.4)
Amount of final bet	1.44(1.7)	0.95(1.4)	1.02 (1.5)

Percentages may not add up to 100% because of missing values or rounding. There were no significant differences in any of the above characteristics between conditions.

We also conducted a linear regression analyses to assess the extent to which participants’ post-cue exposure craving scores predicted wager amount in the final bet after controlling for gender, age of first gamble and days gambled in a typical week. As Table 2 reveals, the overall model was statistically significant, *F*(4,73) = 4.12, *p* = .005, adjusted *R*^2^ =.14, and post-cue exposure craving was the only significant predictor of the amount wagered in the final bet (b = .42, *p* < .001). This result indicates that greater craving reported by participants was associated with higher wagers in the final bet even when controlling for other factors.

**Table 2.** Summary of linear regression analysis

DV	IV	*β*	*T*-value
Final bet	Post-CE GUS score	.42	4.08^***^
	Gender	−.15	−1.33
	Age of 1st gamble	−.14	−1.25
	Days gambled in a typical week	−.06	−.52

Overall *F*(4,73) = 4.12,*p* = .005; ^***^ *p* < .001; Abbreviations: DV = dependent variable; IV = independent variable; CE = cue exposure; GUS = Gambling Urge Scale.

## Discussion

Given previous research on the prevalence of gambling among university students, the impact of wins and losses on craving, and the limitations of previous research on the validity of the GUS, we designed the present study to assess(a) the impact of wins and losses in blackjack on craving using the GUS and (b) the extent to which GUS craving is associated with short-term future gambling behavior. We found that playing computerized blackjack for points increased craving reported by participants regardless of winning or losing. In addition, craving following playing blackjack was the only significant predictor of a wager amount of real money in a betting task when analyzed with gender and gambling history characteristics. This suggests that the GUS can be used to make *a priori* predictions regarding short-term future gambling.

The increase in craving after cue exposure, in this case playing computerized blackjack for points, is consistent with other studies that assessed exposure to photographs, imagery scripts and videos depicting gambling scenarios (Ashrafioun et al., 2012; Sodano & Wulfert, 2010; Wulfert et al., 2009). This study extends previous findings such that this is the first study to utilize a multi-item measure of craving to assess the effects of wins and or losses on craving (Ashrafioun & Rosenberg, 2012; Rosenberg, 2009). However, that we did not find a difference in craving between the Win condition and Lose condition is inconsistent with previous research.

Although this difference may be the result of employing a multi-item measure of craving instead of a single-item measure, there are several other potential explanations. One key difference between the current investigation and previous studies is that other investigations employed different types of exposure to gambling-related cues. In our study, participants played computerized blackjack for points in a laboratory whereas, in Kushner et al. (2008), participants actually held playing cards in their hands and were in an environment that had richer visual and auditory gambling cues. Sodano and Wulfert (2010) found stronger urges when watching videos of winning compared to losing; however, Wulfert et al. (2009) contend that actively participating in gambling (as in our study) creates more excitement than passively watching (as in Sodano & Wulfert, 2010). Further research evaluating the way in which outcomes are manipulated is needed to elucidate the relationship between craving and such outcomes of gambling.

Although the adjusted *R*^2^ was relatively small, the current investigation provides support for the relationship between craving and actual gambling behavior. Previous research has shown that self-reported GUS craving is associated with a variety of gambling-related self-report measures (Ashrafioun et al., 2012; Raylu & Oei, 2004; Smith et al., 2010); however, this was the first investigation indicating that GUS craving is associated with gambling *in situ.* Similar to our study, Young and Wohl (2009) found that craving, as measured by the multi-dimensional Gambling Craving Scale, was associated with future gambling. Specifically, they found that craving was associated positively with subsequent persistent gambling despite continued losses in a virtual slots game. Although the current study was conducted in a lab setting, we believe it was greater ecological validity than that reported in Young and Wohl (2009), given that participants in the current report could wager actual money – as opposed to points – in the optional betting task. However, this money, that participants wagered, was their compensation for participating and it is unclear what differences would have occurred if participants wagered their own money that was not provided for compensation. Furthermore, although we included gambling status, frequency of gamble, and age of first gamble, additional studies should control for other factors (e.g., gambling-related cognitive distortions, gambling problem severity) that may account for the variance of the amount wagered in the optional betting task.

In addition to the limitations noted above, there are several others that restrict the generalizability of this study. For example, our sample was recruited from a single university and included mostly low frequency gamblers, most of whom preferred a mode of gambling other than blackjack. In addition, participants’ mean craving post-cue exposure craving was only 2.6 and 2.3 out of 5 in the Win and Lose conditions, respectively. However, given that craving increased across participants and predicted future wagering in a lab setting, we believe that our findings are a conservative estimate of how a sample of problem or pathological gamblers who prefer blackjack as their primary gambling mode would respond in a setting with more gambling-related cues. Nonetheless, future studies that include a broad range of recreational and problem gamblers recruited from multiple universities would increase the generalizability of the current findings. Another limitation is that we did not include a no-exposure control group to compare to those in the Win condition and Lose condition. Therefore, we cannot rule out the possibility that the increase in craving among the participants was the result of completing the GUS twice within a few minutes. These limitations notwithstanding, this study provides several potential avenues to assess further craving as a function of betting outcome and its association with actual gambling behaviors.
